# Alternating 3 different influenza vaccines for swine in Europe for a broader antibody response and protection

**DOI:** 10.1186/s13567-022-01060-x

**Published:** 2022-06-15

**Authors:** Anna Parys, Elien Vandoorn, Koen Chiers, Kristien Van Reeth

**Affiliations:** 1 Laboratory of Virology, Faculty of Veterinary Medicine, Ghent University, Salisburylaan 133, 9820 Merelbeke, Belgium; 2 Laboratory of Veterinary Pathology, Faculty of Veterinary Medicine, Ghent University, Salisburylaan 133, 9820 Merelbeke, Belgium

**Keywords:** Influenza A virus, swine, vaccination, heterologous prime-boost, antibody cross-reactivity, efficacy, commercial swine influenza vaccine

## Abstract

Heterologous prime-boost vaccination with experimental or commercial influenza vaccines has been successful in various animal species. In this study, we have examined the efficacy of alternating 3 different European commercial swine influenza A virus (swIAV) vaccines: the trivalent Respiporc^®^ FLU3 (TIV), the bivalent GRIPORK^®^ (BIV) and the monovalent Respiporc^®^ FLUpan H1N1 (MOV). Five groups of 6 pigs each received 3 vaccinations at 4–6 week intervals in a homologous or heterologous prime-boost regimen. A sixth group served as a mock-vaccinated challenge control. Four weeks after the last vaccination, pigs were challenged intranasally with a European avian-like H1N1 (1C.2.1) swIAV, which was antigenically distinct from the vaccine strains. One heterologous prime-boost group (TIV–BIV–MOV) had higher hemagglutination inhibition (HI) and neuraminidase inhibition antibody responses against a panel of antigenically distinct H1N1, H1N2 and H3N2 IAVs than the other heterologous prime-boost group (BIV–TIV–MOV) and the homologous prime-boost groups (3xTIV; 3xBIV; 3xMOV). Group TIV–BIV–MOV had seroprotective HI titers (≥ 40) against 56% of the tested viruses compared to 33% in group BIV–TIV–MOV and 22–39% in the homologous prime-boost groups. Post-challenge, group TIV–BIV–MOV was the single group with significantly reduced virus titers in all respiratory samples compared to the challenge control group. Our results suggest that the use of different commercial swIAV vaccines for successive vaccinations may result in broader antibody responses and protection than the traditional, homologous prime-boost vaccination regimens. In addition, the order in which the different vaccines are administered seems to affect the breadth of the antibody response and protection.

**Supplementary Information:**

The online version contains supplementary material available at 10.1186/s13567-022-01060-x.

## Introduction

Swine influenza A viruses (swIAVs) of 3 subtypes—H1N1, H1N2 and H3N2—are endemic in swine populations worldwide. These viruses evolve rapidly by the exchange of genome segments, or “genetic reassortment”, and by mutations in the viral surface proteins resulting in “antigenic drift”.

Multiple introductions of human and avian IAVs to swine have resulted in the concurrent circulation of genetically and antigenically distinct virus strains within each subtype. Because swine populations are geographically separated, the prevailing virus strains also differ between different continents and regions. Based on the genetic differences, the H1 subtype is further classified into 3 major H1 lineages designated by the number 1 followed by the letters A, B or C [1]. The classical swine lineage 1A includes viruses with a H1 related to that of the 1918 pandemic H1N1 virus. The 2009 pandemic H1N1 virus (pH1N1) also belongs to this lineage [2]. The human seasonal lineage 1B derived a hemagglutinin (HA) from human seasonal IAVs. A “human-like H1” was introduced in swine in Europe in the mid-1980s [3, 4] and in North America around 2000 [5]. The Eurasian avian lineage 1C circulates only in Europe and Asia. The H1 derives from an avian H1N1 virus that crossed the species barrier from wild ducks to swine in Europe in 1979 [6]. The 3 major lineages are further subdivided into clades, which are denoted by 1–3 digits. All swIAVs of the H3 subtype are derived from a human ancestor IAV and are classified using another system. Here, the number 3 is followed by the decade in which the virus spilled over from humans to swine [7]. Essentially, there are 3 major lineages: the “European human-like H3” (3.1970) [8–10], the “North American human-like H3” (3.1990) [11] and the “novel human-like H3” (3.2010) [12]. As for the H1, there are 3 N1 lineages: N1avian (N1_av_), N1pandemic (N1_pdm_) and N1classical (N1_classical_) [13]. Within the N2 subtype, there are 4 European lineages, which are derived from a human-like H1N2 swIAV (N2_swSC94_), a human-like H3N2 swIAV (N2_swG84_) [14], human seasonal H3N2 (N2_hs_) [13] and a human H3N2 IAV from 2000, which only circulates in Italy (N2_it_) [15]. In North America N2 lineages are derived from 2 separate human–swine transmission episodes [16]: the first in the late 1990s (N2.1998) and the second in the early 2000s (N2.2002).

Vaccination is the most effective tool to control and prevent swIAV infections. Most commercial swIAV vaccines are inactivated, adjuvanted whole-virus vaccines (WIVs) for intramuscular administration [17, 18]. Protection is based on the induction of serum antibodies to the external viral proteins HA and, to a lesser extent, neuraminidase (NA). High antibody titers against HA and NA will, respectively, inhibit virus entry into host cells and prevent viral release from infected cells. Given the genetic and antigenic differences between swIAVs circulating in different continents and regions, vaccines are produced locally. Even within a continent, vaccines from different manufacturers are not standardized with respect to the number of virus subtypes and strains, the nature of the vaccine strains, the antigen dose, and type of adjuvant. In Europe, the first swIAV vaccines were licensed during the late 1980s. These oil-adjuvanted WIVs contained the 2 virus subtypes that were prevalent at that time: H1N1 and H3N2. Nowadays, 1 such bivalent vaccine is still sold under the brand name GRIPORK^®^. A trivalent vaccine, Respiporc^®^ FLU3, includes the H1N2 subtype next to H1N1 and H3N2. It was only licensed in 2010 and has a carbomer adjuvant. It is currently Europe’s most widely used vaccine [18]. Its monovalent counterpart, Respiporc^®^ FLUpan H1N1, only contains a pH1N1 virus strain. It was licensed in 2017 with the purpose to protect against the recently emerged pH1N1 virus. The current commercial swIAV vaccines have been shown to be effective against swIAVs that are related to the vaccine strains. The extent of cross-protection against heterologous swIAV can be limited [19–28] and seems to rely on multiple factors including antigen dose, adjuvant, and genetic/antigenic homology to the vaccine strains [19, 21–23, 26, 29]. Importantly, no vaccine can protect against all the existing swIAV lineages and clades.

The ever-increasing diversity of swIAVs calls for more broadly protective vaccines or vaccination strategies. One such strategy is heterologous prime-boost vaccination, or the use of vaccines based on antigenically distinct strains within a subtype for primary and booster vaccinations. It has been shown to broaden cross-clade immunity within a subtype in humans, ferrets and domestic animals [30–32]. In swine, we have obtained promising results by 3-dose heterologous prime-boost vaccination with experimental monovalent WIVs based on the 3 different European swine H1 lineages (Van Reeth et al., unpublished observations). This strategy has not yet been examined with European commercial swIAV vaccines. The objective of this study was to compare the antibody responses and protection against challenge after vaccination with commercial swIAV vaccines in a homologous or a heterologous prime-boost vaccination regimen.

## Materials and methods

### Viruses and vaccines

Three different European commercial swIAV WIVs were used for immunization. (i) the trivalent vaccine Respiporc^®^ FLU3 (Ceva, Germany, lot no. 2621118), further referred to as TIV, (ii) the bivalent vaccine GRIPORK^®^ (Hipra, Spain, lot no. 12Q2-1), further referred to as BIV and (iii) the monovalent vaccine Respiporc^®^ FLUpan H1N1 (Ceva, Germany, lot no. 0370819B), further referred to as MOV. Table 1 shows the vaccine strains, antigen doses and adjuvants for each vaccine. The antigen doses cannot be directly compared between vaccines because they are measured and expressed in a different way.

**Table 1:** **Swine influenza virus vaccines included in the study and their composition**.

Vaccine	Influenza virus strains	Subtype	HA clade	NA lineage	Adjuvant	Vaccine dose (mL)	Antigenic content per dose
Respiporc^®^ FLU3 (TIV)	A/swine/Haselünne/IDT2617/2003	H1N1	1C.2.2	N1_av_	Carbomer	2	≥ 10.22 log_2_ GMNU
	A/swine/Bakum/1832/2000	H1N2	1B.1.2.1	N2_swSC94_			≥ 12.34 log_2_ GMNU
	A/swine/Bakum/IDT1769/2003	H3N2	3.1970.1	N2_swG84_			≥ 10.53 log_2_ GMNU
GRIPORK^®^ (BIV)	A/swine/Olost/84	H1N1	1C.1.2-like	N1_av_	Oil	2	7.48 log_10_ EID_50_
	A/Port Chalmers/1/73	H3N2	3.1970.1	N2_swG84_			7.40 log_10_ EID_50_
Respiporc^®^ FLUpan H1N1 (MOV)	A/Jena/VI5258/2009	pH1N1*	1A.3.3.2	N1_pdm_	Carbomer	1	≥ 16 HAU

GMNU, geometric mean of neutralizing units induced in guinea pigs after 2 immunizations with 0.5 mL of the vaccine; EID_50_, 50% egg infectious dose before inactivation; HAU, hemagglutinating units.

^*^pH1N1, 2009 pandemic H1N1 virus.

**Figure 1:**
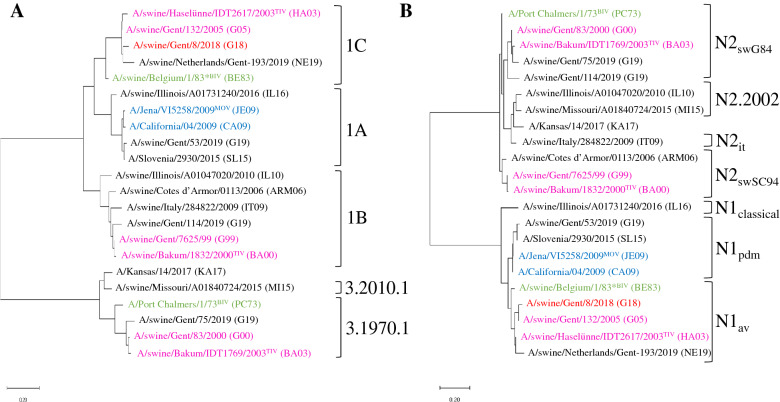
**Maximum likelihood phylogenetic tree of influenza A viruses included in the study.** The phylogenetic tree is based on the hemagglutinin 1 (HA1) (**A**) and neuraminidase (NA) (**B**) amino acid sequences of the influenza A virus strains used for vaccination, challenge and serological assays. The vaccine strains and strains representative for the vaccine strains are colored in: magenta, TIV (Respiporc^®^ FLU3); green, BIV (GRIPORK^®^); blue, MOV (Respiporc^®^ FLUpan H1N1). The challenge virus is colored in red. The lineage of the HA and NA is indicated. H1 and H3 lineages include: 1A, classical swine H1; 1B, human-like H1; 1C, avian-like H1; 3.1970.1, European human-like H3; 3.2010.1, novel human-like H3. N1 and N2 lineages include: N1_av_, N1avian; N1_pdm_, N1pandemic; N1_classical_, N1classical; N2_swSC94_, H1N2 A/swine/Scotland/410440/94-like N2; N2_swG84_, H3N2 A/swine/Gent/1/84-like N2; N2_it_, N2 introduced from humans to Italian swine in 2000; N2.2002, N2 introduced from humans to North American swine in the early 2000s. *For A/swine/Olost/84, the avian-like H1N1 vaccine strain in BIV, no gene sequences are made publicly available. We therefore selected an avian-like H1N1 (1C.2.1-like) swIAV from 1983 as a substitute for the genetic and serological investigations.

Because homologous vaccine strains were not available, except for A/Port Chalmers/1/73, we selected representative virus strains that are genetically closely related to the vaccine strains (Figure 1) for use in serological assays. For A/swine/Olost/84, no gene sequences are made publicly available. Therefore, an avian-like H1N1 (1C.1.2-like) swIAV from 1983 was selected as a substitute for the genetic (Figure 1) and serological investigations. The challenge virus was A/swine/Gent/8/2018 (G18) (1C.2.1), a swIAV that is representative for avian-like H1N1 swIAVs, the predominant swIAV lineage in Europe [7, 15, 33–35]. We obtained European swIAV A/swine/Côtes d’Armor/0113/2006 and corresponding swine serum from the French agency for food, environmental and occupational health and safety (ANSES) and A/swine/Italy/284822/2009 and corresponding swine serum from the Istituto Zooprofilattico Sperimentale della Lombardia e dell’Emilia Romagna “Bruno Ubertini” (IZSLER). The North American swIAVs and corresponding swine sera were obtained from the U.S. Department of Agriculture-Agricultural Research Service (USDA). Human seasonal IAVs and corresponding ferret sera were obtained from the Francis Crick Institute (London, UK).

The coding sequences of the HA1 and NA segments of the viruses are available in GenBank [36] or GISAID [37]. To determine the genetic relationship between the viruses, protein sequences were aligned with ClustalW, and the P sequence values and P all antigenic site values for the HA1 and NA were determined (Additional files 1, 2, 3 and 4) [38–43]. P sequence value is defined as the number of amino acid (aa) substitutions in the HA1 or NA divided by the total number of aa. P all antigenic site value is the number of aa substitutions in the antigenic sites divided by the total number of aa in the antigenic sites. Based on the protein sequences, a maximum likelihood tree was constructed (Figure 1) using MEGA7 [44] using the Jones-Taylor-Thornton substitution model with a gamma (Γ) distribution among site rate. Branch length is proportional to genetic distance.

### Experimental design

Thirty-six 5-week-old pigs were obtained from a herd free of IAVs. The pigs were assigned to 6 different groups and housed together in a BSL2 isolation facility with 6 pens and HEPA-filtered air. At arrival, serum samples of all individual pigs were confirmed to be seronegative in a competitive anti-IAV nucleoprotein enzyme-linked immunosorbent assay (ID-VET) and in HI assays against H1N1, H1N2 and H3N2 influenza viruses that are representative for swIAVs currently circulating in Europe. To examine the effect of heterologous prime-boost vaccination with European commercial swIAV vaccines, pigs were vaccinated as shown in Table 2. Five groups were vaccinated 3 times at a 4- and 6-week interval respectively with 3 different vaccines (heterologous prime-boost, 2 groups) or with the same vaccine (homologous prime-boost, 3 groups). The sixth group was mock-vaccinated with phosphate buffered saline (PBS) and 20% commercial oil-in-water adjuvant (Emulsigen, MVP Laboratories, NE, USA). The latter served as a mock-vaccinated challenge control. Vaccines were administered by deep intramuscular injection into the neck. Four weeks after the last vaccination, groups were moved to individual isolation rooms and challenged intranasally with 7.0 log_10_ tissue culture infectious doses 50% (TCID_50_)/5 mL of G18. Serum samples were collected at arrival and 4 weeks after each vaccination. Nasal swabs were collected at 0 and 3 days post-challenge to determine nasal shedding of virus. Pigs were humanly euthanized at 3 days post-challenge to collect lung and trachea samples for determination of microscopic and macroscopic lesions and viral titers.

**Table 2:** **Experimental design of the study**.

Prime-boost regimen	Group	Vaccinations			Challenge (week 14)
		First (week 0)	Second (week 4)	Third (week 10)	
n.a	Mock-vaccinated challenge control	PBS	PBS	PBS	A/swine/Gent/8/2018
Homologous	3×TIV	TIV	TIV	TIV	
	3×BIV	BIV	BIV	BIV	
	3×MOV	MOV	MOV	MOV	
Heterologous	TIV–BIV–MOV	TIV	BIV	MOV	
	BIV–TIV–MOV	BIV	TIV	MOV	

n.a.: not applicable; PBS: phosphate buffered saline; TIV: Respiporc® FLU3; BIV: GRIPORK®; MOV: Respiporc® FLUpan H1N1.

### Serological investigations

All serum samples were examined in HI and enzyme-linked lectin assays (ELLA) to determine HI and neuraminidase inhibition (NI) antibody titers against the H1N1, H1N2 and H3N2 viruses used for immunization. Sera collected 4 weeks after the second (week 8) and third (week 14) vaccination were also examined in HI and ELLA against 12 and 7 viruses respectively, representing swIAVs currently circulating in Europe and North America and IAVs circulating in the human population (Figure 1) [1, 7, 13, 15, 33, 45]. HI and ELLA were performed according to standard procedures and starting dilutions were 1:10 [46, 47]. HI and NI titers represent the reciprocal of the highest serum dilution that respectively inhibited hemagglutination of 4 hemagglutinating units of virus or that reduced NA activity by 50%.

For the HI assays, serum samples were inactivated at 56 °C for 30 min and subsequently treated with receptor destroying enzyme (RDE) at 37 °C for 18 h; 50% turkey erythrocytes were then added to eliminate non-specific HI factors. Prior to the ELLA, heat inactivated serum samples were pre-treated with RDE at 37 °C for 18 h.

### Virus titration

We determined virus titers in nasal swabs and in 20% tissue homogenates of the trachea and a pooled sample of the apical, cardiac and diaphragmatic lobes of the left and right lung. Swabs from both nostrils were suspended in 1 mL of PBS supplemented with antibiotics and vigorously shaken for 1 h at 4 °C. Tissue samples were processed to 20% tissue homogenates in PBS with Ca^2+^ and Mg^2+^ supplemented with antibiotics. For both nasal swab samples and 20% tissue homogenates the medium was clarified by centrifugation and the supernatant was used for virus titration.

Virus titrations were performed in Madin–Darby Canine Kidney (MDCK) cell cultures. Ten-fold serial dilutions were made and inoculated on 96-well plates using 4 wells/dilution. MDCK cells were observed daily for cytopathic effect until 7 days post-inoculation. Virus titers were calculated using the Reed and Muench method [48] and expressed as log_10_ TCID_50_/g tissue or /100 mg nasal secrete.

### Lesion scores of trachea and lungs

At necropsy, we determined the percentage of lung affected with purple-red consolidation typical of swIAV infection. The percentage of the surface affected with pneumonia was visually estimated for each lung lobe, and a total percentage for the entire lung was calculated, based on weighted proportions of each lobe to the total lung volume [49]. Tissue samples from the trachea and right cardiac lung were fixed in 4% buffered formalin for histopathologic examination. Tissues were processed by routine histopathological procedures and slides were stained with hematoxylin and eosin. Microscopic lesions were scored as previously described by a veterinary pathologist blinded to treatment groups [50].

### Statistical analysis

Lesion scores and log_10_-transformed virus titers were compared among groups using the Mann–Whitney U test or the Kruskall–Wallis test followed by the Dunn’s test. A *p*-value of ≤ 0.05 was considered significant. Virus titers below the detection limit (1.7 log_10_ TCID_50_) were assigned a value of 0.85 log_10_ TCID_50_.

## Results

### Genetic relatedness between test viruses

To visualize the genetic relationship between the viruses used for vaccination, challenge and serological assays, we constructed phylogenetic trees of the HA1 and NA aa sequences (Figure 1). Additional files 1, 2, 3 and 4 show the P sequence values and P all antigenic site values of the HA1 and NA of the virus strains. As shown in Figure 1, the vaccine strains and their representative virus strain used for serology are genetically closely related to each other. The virus strains included in the 3 swIAV vaccines were genetically distant from each other. Pairwise comparisons of the 4 H1 vaccine strains (Additional file 1) resulted in P sequence values between 0.141 and 0.296 and P all antigenic site values between 0.240 and 0.660. Between the 2 H3 vaccine strains (Additional file 2) the P sequence value was 0.137 and the P all antigenic site value 0.225. The challenge virus G18 is a recent avian-like H1N1 swIAV that is genetically distinct from the H1 vaccine strains (Figure 1). The P sequence values between G18 and the 4 H1 vaccine strains varied between 0.083 and 0.294. The P all antigenic site values varied between 0.180 and 0.640. To analyze the breadth of the antibody response against the HA and NA, sera were tested against heterologous swine and human IAVs in HI (12 strains) and ELLA (7 strains). Comparisons of the H1 vaccine strains and the test strains yielded P sequence values between 0.034 and 0.309 and P all antigenic site values between 0.080 and 0.680. Comparisons of the H3 gave P sequence values between 0.082 and 0.231 and P all antigenic site values between 0.175 and 0.400. For the NA of the virus strains, the P sequence values were up to 3 times lower compared to the values for HA. On the other hand, the P all antigenic site values were up to 9 times lower. This implies that for this study more substitutions are located in the antigenic sites of HA compared to NA.

### Serum antibody response against vaccine strains following a homologous or heterologous prime-boost vaccination regimen

**Figure 2:**
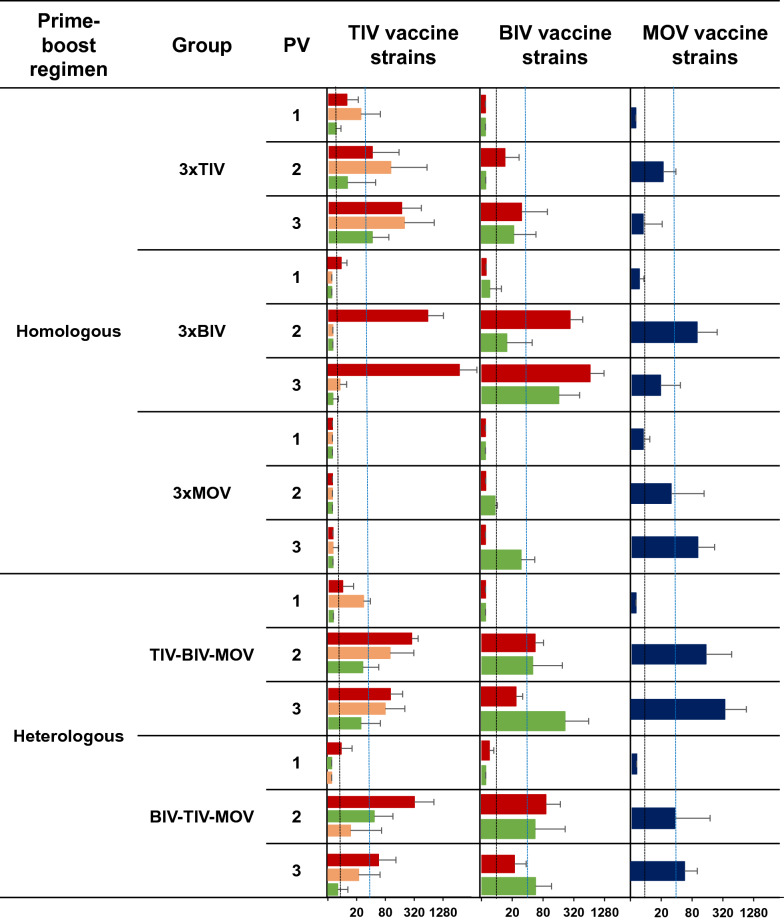
**Hemagglutination inhibition (HI) antibody titers against the vaccine strains post-vaccination (PV) 1, 2 and 3.** Geometric mean HI antibody titers and the standard deviation were determined 4 weeks post each vaccination. Black dotted lines indicate the detection limit (10), blue dotted lines indicate the seroprotective threshold (40). The colors of the bars are based on the HA lineage of the vaccine strains. Blue, classical swine H1 (1A.3.3.2); orange, human-like H1 (1B); green, avian-like H1 (1C); dark red, European human-like H3 (3.1970.1). TIV, Respiporc^®^ FLU3; BIV, GRIPORK^®^; MOV, Respiporc^®^ FLUpan H1N1. Representative virus strains that are genetically closely related to the vaccine strains were used in the HI assay (Figure 1).

**Figure 3:**
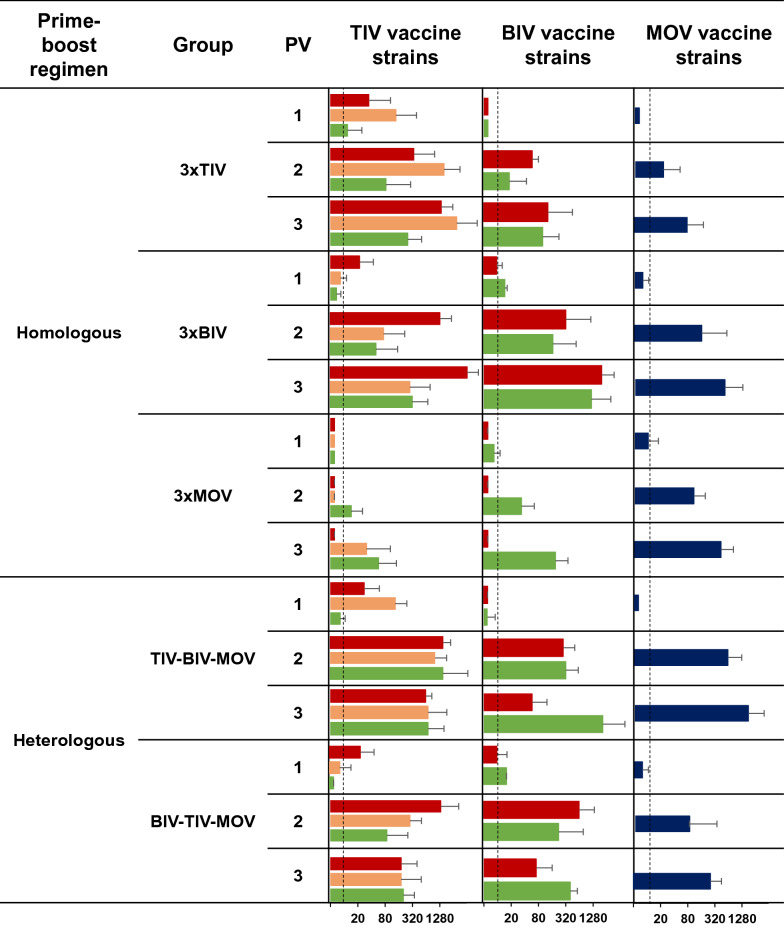
**Neuraminidase inhibition (NI) antibody titers against the vaccine strains post-vaccination (PV) 1, 2 and 3.** Geometric mean NI antibody titers and the standard deviation were determined 4 weeks post each vaccination. Black dotted lines indicate the detection limit (10). The colors of the bars are based on the NA lineage of the vaccine strains. Blue, N1pandemic; orange, H1N2 A/swine/Scotland/410440/94-like N2; green, N1avian; dark red, H3N2 A/swine/Gent/1/84-like N2. TIV, Respiporc^®^ FLU3; BIV, GRIPORK^®^; MOV, Respiporc^®^ FLUpan H1N1. Representative virus strains that are genetically closely related to the vaccine strains were used in the NI assay (Figure 1).

HI and NI titers against the vaccine strains were determined using sera collected 4 weeks post each vaccination (weeks 4, 8 and 14). Figures 2 and 3 show respectively the geometric mean HI and NI antibody titers and their standard deviation against each of the vaccine strains in each of the 5 vaccinated groups. An HI antibody titer of 40 or more against a particular influenza virus is considered seroprotective [51]. All pigs were negative for IAV antibodies at the start of the experiment, and the challenge control group remained seronegative throughout the experiment.

Four weeks after the first vaccination (PV 1), HI antibody titers were undetectable or low in all vaccinated groups. In the homologous prime-boost groups, HI antibody titers against the homologous vaccine strains increased by 2- to 36-fold after the second vaccination (PV 2). After 3 vaccinations, HI titers of  ≥ 40 were obtained against all homologous vaccine strains, except against the TIV H1N1 (1C.2.1) vaccine strain. At this timepoint, group 3xBIV also had HI titers of  ≥ 40 against the TIV H3N2 (3.1970.1) vaccine strain and the MOV pH1N1 (1A.3.3.2) vaccine strain.

Unlike the homologous prime-boost groups, both heterologous prime-boost groups had HI antibody titers above the detection limit against all 6 vaccine strains after the second vaccination (PV 2). Moreover, for group TIV–BIV–MOV, HI titers were  ≥ 40, except for the TIV H1N1 (1C.2.1) vaccine strain. After the third vaccination (PV 3), a further increase in HI antibody titers was only observed against the MOV pH1N1 (1A.3.3.2) and the BIV H1N1 (1C.1.2-like) vaccine strains.

Four weeks after the first vaccination (PV 1), NI antibody titers were mainly present against the vaccine strains included in the administered vaccine (Figure 3). After the second vaccination (PV 2), homologous prime-boost groups had NI antibodies against all 6 vaccine strains, except for group 3xMOV. The latter group only had NI antibody titers against N1 vaccine strains (N1_av_ and N1_pdm_). The third vaccination (PV 3) resulted in a two- to sevenfold increase in NI antibody titers.

Like the homologous prime-boost groups, both heterologous prime-boost groups had NI antibodies against all 6 vaccine strains 4 weeks after the second vaccination (PV 2). After 3 vaccinations, NI antibody titers in group TIV–BIV–MOV were 2-to 80-fold higher than in the homologous prime-boost groups, except against the TIV H1N2 vaccine strain (N2_swSC94_) and both H3N2 vaccine strains (N2_swG84_). NI antibody titers were generally higher in group TIV–BIV–MOV than in group BIV–TIV–MOV.

In summary, 3 homologous vaccine administrations were needed to induce HI titers of  ≥ 40 against the homologous vaccine strains. On the other hand, for group TIV–BIV–MOV, 2 vaccine administrations succeeded to induce HI titers of  ≥ 40 against 5 out of the 6 vaccine strains, but titers hardly improved following the third (MOV) vaccination. After 2 vaccinations, all groups had NI antibody titers against all 6 vaccine strains, except for 3xMOV.

### Serum antibody response against antigenically diverse H1N1, H1N2 and H3N2 IAVs of swine and humans

Four weeks after the second (PV 2) and third vaccination (PV 3), we determined HI antibody titers of the individual pigs against a panel of 18 IAVs. NI antibody titers were determined against a panel of 13 IAVs. The test viruses included the vaccine strains and strains that are representative for swIAVs currently circulating in Europe and North America, and human IAVs currently circulating worldwide.

**Figure 4:**
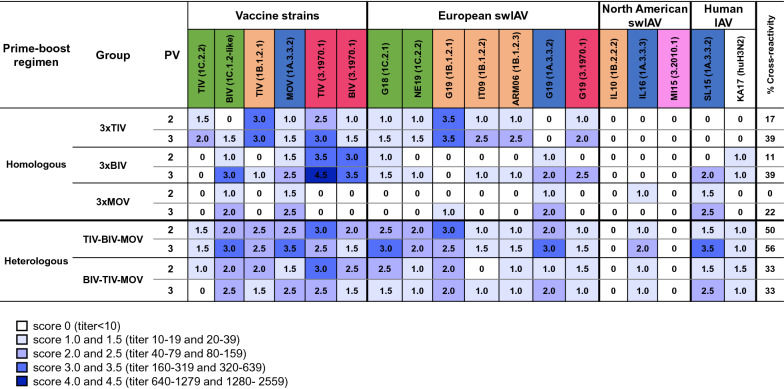
**Hemagglutination inhibition (HI) titer scores against vaccine strains and antigenically distinct influenza A virus strains.** Titers were determined 4 weeks after the second and third vaccination (PV 2 and 3). The colors of the test viruses are based on the HA lineage. Blue, classical swine H1 (1A); orange, human-like H1 (1B); green, avian-like H1 (1C); dark red, European human-like H3 (3.1970.1); magenta, novel human-like H3 (3.2010.1). The HA clade is mentioned between brackets. Antibody titers were given a score from 0 to 4.5; the scoring system is explained below the Figure. The scores are means of the scores of the individual pigs of each group; they are converted to a heatmap. The % cross-reactivity indicates the percentage of virus strains against which an HI titer score ≥ 2.0 was achieved. See Figure 1 for full names of IAVs and the vaccine names.

**Figure 5:**
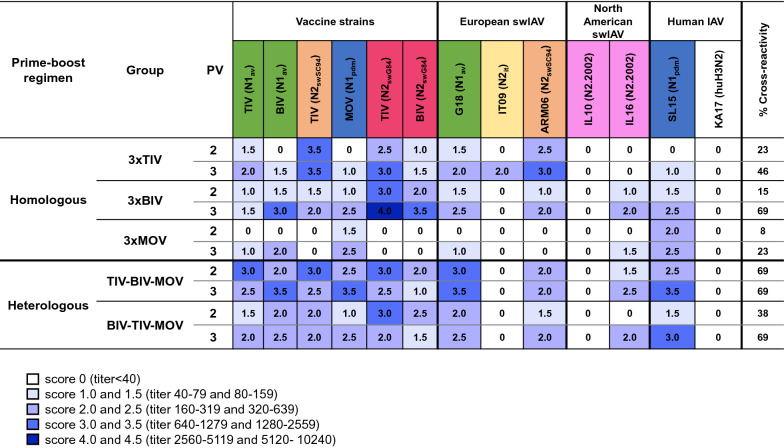
**Neuraminidase inhibition (NI) titer scores against vaccine strains and antigenically distinct influenza A virus strains.** Titers were determined 4 weeks after the second and third vaccination (PV 2 and 3). The colors of the test viruses are based on the NA lineage. Blue, N1pandemic; orange, H1N2 A/swine/Scotland/410440/94-like N2; green, N1avian; dark red, H3N2 A/swine/Gent/1/84-like N2; yellow, N2_it_; magenta, N2.2002. The NA virus lineage is mentioned between brackets. Antibody titers were given a score from 0 to 4.5; the scoring system is explained below the Figure. The scores are means of the scores of the individual pigs of each group; they are converted to a heatmap. The % cross-reactivity indicates the percentage of virus strains against which a NI titer score ≥ 2.0 was achieved. See Figure 1 for full names of IAVs and the vaccine names.

To compare HI antibody titers between vaccinated groups, we used a scoring system (scores 0–4.5, see Figure 4) that is based on the accepted seroprotective threshold for seasonal influenza in adults [51] and data from vaccination-challenge studies in pigs [52]. In influenza-naïve pigs, an HI titer of 160–320 is required to protect 50% of the pigs against infection upon challenge in vaccination-challenge studies. For NI antibody titers we implemented a different scoring system since NI titers (Figure 5) were higher than HI titers. For each vaccinated group, we also calculated the number of virus strains with HI or NI titer scores ≥ 2.0. This number was converted to a percentage and expressed as % cross-reactivity.

Four weeks after the second vaccination (PV 2), homologous prime-boost groups had only 0–17% cross-reactivity and HI titer scores ≥ 2.0 were mainly found against the homologous vaccine strains. After the third vaccination (PV 3), cross-reactivity scores increased to 22–39%. All homologous prime-boost groups had an HI titer score ≥ 2.0 against the homologous vaccine strains and closely related strains. Additionally, group 3xTIV had HI titer scores ≥ 2.0 against IT01 (1B.1.2.2) and ARM06 (1B.1.2.3), both swIAVs from another clade than the TIV H1N2 (1B.1.2.1) vaccine strain. For group 3xBIV, additional HI titer scores ≥ 2.0 were observed against pH1N1 (1A.3.3.2) viruses and the recent H3N2 virus G19 (3.1970.1). Three MOV administrations resulted in an HI titer score ≥ 2.0 against the H1N1 BIV (1C.1.2-like) vaccine strain. No group had HI titer scores ≥ 2.0 against challenge virus G18 (1C.2.1).

For heterologous prime-boost group TIV-BIV-MOV, 2 vaccinations already resulted in 50% cross-reactivity. Only against 2 North American swIAVs, IL10 (1B.2.2.2) and MI15 (3.2010.1), antibodies were completely lacking. Heterologous prime-boost group BIV–TIV–MOV had a similar antibody response pattern, but HI titer scores were up to twofold lower and the cross-reactivity score was only 33%. After the second vaccination, both heterologous prime-boost groups already had an HI titer score ≥ 2.0 against challenge virus G18 (1C.2.1). After 3 vaccinations, the cross-reactivity score increased to 56% for group TIV–BIV–MOV and HI titer scores ≥ 2.0 were obtained against viruses from the classical swine lineage (1A). For group BIV-TIV-MOV the cross-reactivity score remained 33%. This was due to an increase in HI titer scores against viruses from clade 1A.3.3.2 but decreased scores against other tested viruses, including G18 (1C.2.1).

Four weeks after the second vaccination (PV 2), homologous prime-boost groups had NI titer scores ≥ 1.5 against the homologous vaccine strains and cross-reactivity scores between 8 and 23% (Figure 5). After the third vaccination (PV 3), the NI titer scores increased up to twofold and cross-reactivity scores varied between 23 and 69%.

Heterologous prime-boost group TIV–BIV–MOV already had 69% cross-reactivity following 2 vaccinations, compared to 33% in group BIV–TIV–MOV. Four weeks after the third vaccination, the % cross-reactivity score remained 69% for group TIV–BIV–MOV and increased to 69% for group BIV–TIV–MOV, with mainly higher scores against N1 viruses (N1_av_ and N1_pdm_).

Thus, after the second heterologous vaccination, group TIV-BIV-MOV already had equal or higher HI and NI cross-reactivity scores than the BIV-TIV-MOV group, or than the 3-dose homologous prime-boost groups.

### Protection against challenge

To evaluate protection against avian-like H1N1 swIAV G18, pigs were challenged 4 weeks after the last vaccination and euthanized 3 days post-challenge to examine virus titers in the respiratory tract and macroscopic and microscopic lesions.

**Figure 6:**
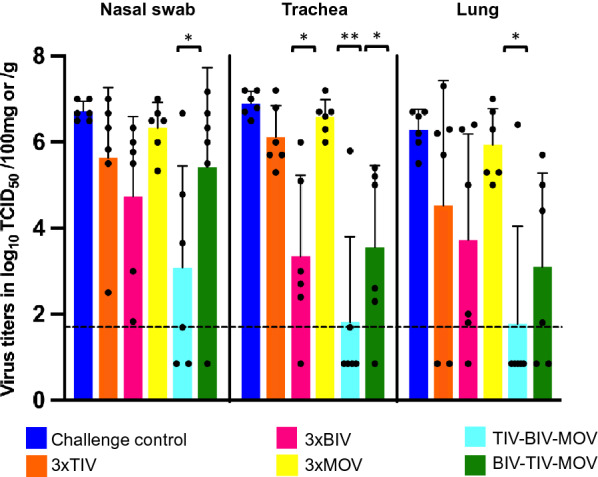
**Virus titers in the respiratory tract 3 days post-challenge with A/swine/Gent/8/2018.** The black dots represent virus titers of individual pigs, the bars represent the mean virus titer ± the standard deviation. Each color represents a different group. Dotted lines indicate the detection limit. Virus titers in nasal swabs are expressed in log_10_ TCID_50_/100 mg nasal secrete, virus titers in the trachea and lung are expressed in log_10_ TCID_50_/g tissue. Significant reductions of virus titers as compared with the challenge control group are indicated by asterisks. *p < 0.05; **p < 0.01.

Figure 6 shows the individual and mean virus titers and the standard deviation in the nasal swabs, the trachea and the lung samples of the challenge control group and vaccinated groups. In the challenge control group, G18 was isolated from the respiratory tract samples of all pigs and virus titers ranged between 10^5.5^ and 10^7.2^ TCID_50_/g or /100 mg. Homologous prime-boost group 3xMOV was not protected and had high virus titers, similar to those in the challenge control group, in all respiratory tract samples. Compared to the challenge control group, group 3xTIV had reduced mean virus titers in nasal swabs, trachea and lung samples, and no virus could be detected in the lungs of 2 out of 6 pigs, but the reductions in mean titers were not significant. In contrast, group 3xBIV had significantly reduced virus titers in the trachea as compared to the control group (*p* = 0.029). In addition, virus was undetectable in 1 trachea sample and 1 lung sample. Similar to group 3xBIV, heterologous prime-boost group BIV–TIV–MOV had significantly reduced virus titers in the trachea compared to the challenge control group (*p* = 0.018). Two pigs were almost completely protected: 1 pig only had virus in the nasal swabs and another pig only had a low virus titer in the trachea. Overall, protection was most pronounced in heterologous prime-boost group TIV–BIV–MOV, with significantly reduced mean virus titers in all tissues compared to the challenge control group (*p* ≤ 0.048) and in the trachea and lungs compared to 3xMOV (*p* ≤ 0.022). In addition, 5 of the 6 pigs tested negative for virus in the lungs. Taken together, both heterologous prime-boost groups had significantly reduced mean virus titers in the trachea and lungs (*p* ≤ 0.017) compared to the homologous prime-boost groups.

Macroscopic lung lesions and histopathological findings are summarized in Table 3. The challenge control group had an average macroscopic lung lesion percentage of 5.2. In the homologous prime-boost groups, macroscopic pneumonia was less severe in groups 3xTIV and 3xBIV (2.0% and 1.8%) than in the challenge control group and group 3xMOV (3.2%). This was consistent with the lower viral loads in the respiratory tract. In the heterologous prime-boost groups (TIV-BIV-MOV and BIV-TIV-MOV) macroscopic lung lesions were virtually lacking and the average macroscopic lung lesion percentages were lower than 1.0.

**Table 3:** **Trachea and lung pathology after challenge**.

Vaccine group	% macroscopic pneumonia ± SD (number of pigs with lesions)	Microscopic lesion score ± SD (number of pigs with lesions)			
		Cardiac lung			Trachea
		IPAW	PBLC	Neutro’s	
Mock-vaccinated challenge control	5.2 ± 5.5 (5)	0.3 ± 0.5 (2)	1.0 ± 0.6 (5)	0.3 ± 0.5 (2)	0.3 ± 0.5 (2)
3xTIV	2.0 ± 2.5 (5)	1.6 ± 0.9 (4)	1.6 ± 0.6 (6)	1.3 ± 0.8 (5)	0.8 ± 0.4 (5)
3xBIV	1.8 ± 4.1 (2)	0.4 ± 0.7 (2)	1.3 ± 1.2 (4)	0.7 ± 1.2 (2)	0.5 ± 0.6 (3)
3xMOV	3.2 ± 4.7 (4)	0.3 ± 0.8 (1)	1.1 ± 1.0 (4)	0.3 ± 0.8 (1)	0.3 ± 0.5 (2)
TIV–BIV–MOV	0.6 ± 1.2 (2)	0.0	0.8 ± 1.0 (3)	0.0	0.0
BIV–TIV–MOV	0.1 ± 0.2 (1)	0.0	0.7 ± 0.8 (3)	0.2 ± 0.4 (1)	0.0

TIV: Respiporc® FLU3; BIV: GRIPORK®; MOV: Respiporc® FLUpan H1N1.

Macroscopic pneumonia: the percentage of the surface affected with pneumonia was estimated visually for each lobe and the total percentage for the entire lung was calculated, based on weighted proportions of each lobe to the total volume. Microscopic lung lesion scores are based on the severity of 3 parameters: (1) IPAW: epithelial damage in intrapulmonary airways (0–3), (2) PBLC: peribronchiolar lymphocytic cuffing (0–3), (3) Neutro’s: neutrophil exudation in bronchioles and alveoli (0–2); Microscopic tracheal lesion scores are based on the severity of epithelial damage (0–2).

In the challenge control group, microscopic lesions were mild and mainly consisted of peribronchiolar lymphocytic cuffing. Epithelial damage in the lungs and trachea and neutrophil exudation were observed in only 2 out of the 6 pigs. Microscopic lesions in the homologous prime-boost groups were similar as in the challenge control group. In the heterologous prime-boost groups (TIV–BIV–MOV and BIV–TIV–MOV), there was minimal peribronchiolar lymphocytic cuffing and no neutrophil exudation (except for 1 pig) or epithelial damage in the lungs or trachea. There were no significant differences in macroscopic lesion scores and histopathological findings among groups.

Despite the inclusion of an avian-like H1N1 vaccine strain in both TIV and BIV, 3 homologous vaccinations failed to induce significant protection against G18 challenge, except for group 3xBIV in the trachea compared to the challenge control group. On the other hand, heterologous TIV-BIV-MOV vaccination induced significant protection in all tissues compared to the challenge control group and in the trachea and lungs compared to 3xMOV and although not significant, the former group also had reduced respiratory tract pathology.

## Discussion

Heterologous prime-boost vaccination with experimental [52–55] or commercial influenza vaccines [30–32, 56, 57] is a proven strategy to broaden antibody responses and protection in various animal species. Our study is the first to demonstrate that heterologous prime-boost vaccination with 3 different European commercial swIAV vaccines can broaden the immune response against both HA and NA as compared to the traditional homologous prime-boost regimen. Furthermore, we show improved protection against challenge with a heterologous avian-like H1N1 swIAV that circulated in the European swine population at least 15 years after the avian-like H1N1 vaccine strains.

A previous heterologous prime-boost study with commercial North American swIAV vaccines by Li et al. [31] also demonstrated a more complete protection against heterologous H1N1 as well as H3N2 challenge. Apart from the different vaccines and challenge virus strains, the experimental design of the former study differed from ours in that they used 2 instead of 3 vaccinations. We examined HI as well as NI antibody responses against a large panel of antigenically diverse IAVs from swine and humans after each vaccination. In contrast to homologous prime-boost vaccination, 2 vaccinations in a heterologous prime-boost regimen, TIV followed by BIV, were sufficient to induce mean HI antibody titers above the seroprotective threshold of 40 (HI titer score ≥ 2) against a recent pH1N1 (1A.3.3.2) and H1N1 (1C.2) swIAVs, both representative for the predominant swIAV lineages in Europe [35]. Following challenge with G18 (1C.2.1), only heterologous prime-boost group TIV-BIV-MOV had significantly reduced virus titers in all parts of the respiratory tract compared to the challenge control group. Whether pigs would be protected against heterologous challenge with swIAVs of other subtypes or lineages remains unclear. Based on the HI antibody response (HI titer score ≥ 2), we expect at least partial protection against currently circulating European H1N2 (1B.1) and H3N2 (3.1970.1) swIAVs in group 3xTIV, against pH1N1 (1A.3.3.2) IAVs and H3N2 (3.1970.1) swIAVs in group 3xBIV and against pH1N1 (1A.3.3.2) IAVs in group 3xMOV. Currently circulating H1N1 (1C.2) swIAVs, in contrast, appear to be antigenically too distant from the H1N1 (1C) vaccine strains to be covered. Although group TIV-BIV-MOV had an HI titer score of only 1.5 against currently circulating European H1N2 (1B.1) and H3N2 (3.1970.1) swIAVs, this heterologous prime-boost group combined the antibody profiles of the 3 homologous prime-boost groups together with HI titer scores ≥ 2 against H1N1 (1C) swIAVs. On the other hand, consistent with previous swIAV vaccination studies [21, 52], no combination of 2 or 3 vaccines induced an HI titer score ≥ 2 against recent human H3N2 IAVs nor North American swIAVs. The single exception were the antibodies against the γ-H1N1 swIAV IL16 (1A.3.3.3) in group TIV-BIV-MOV.

In our experience, (Chepkwony et al., unpublished observations) (Van Reeth et al., unpublished observations), 2 poorly understood factors are of major importance for the breadth of the anti-HA antibody response following heterologous prime-boost vaccination: (i) the genetic and antigenic relationship between the sequentially administered vaccine strains and (ii) the order of vaccine administration [31, 54, 58] Although it was not the main aim of our study to investigate these factors, our results seem to confirm their importance. It is believed that the antigens for the primo- and booster vaccination need to be sufficiently antigenically distinct, while still sharing conserved epitopes [59]. This way, the immune system will react with a recall response against these conserved epitopes upon the booster vaccination(s). The optimal genetic and antigenic distance is unknown and cannot be deduced from our study. The relevance of the order of vaccine administration was clearly demonstrated by the superior antibody titers and protection in group TIV-BIV-MOV compared to group BIV-TIV-MOV. As previously described by Li et al. [31], this might be explained by the fact that the first encountered antigens in TIV-BIV-MOV were genetically more closely related to the challenge virus than those in BIV-TIV-MOV. The importance of the order of vaccine administration might be explained by the concept of “back-boosting”. This concept states that the antibody response is favored by the first encountered antigens, without diminishing the immune response to the subsequently encountered antigens [60].

In line with previous vaccination studies [19, 21, 22, 24–26, 61], homologous vaccine administrations (3xTIV, 3xBIV, 3xMOV) mainly induced HI antibodies against the viruses included in the administered vaccine or closely related strains (P sequence values 0.034–0.093). A study published in 1999 [62] reported a loss of antigenic cross-reactivity between the BIV H3N2 vaccine strain and the circulating swIAVs and the authors recommended to update the H3N2 component in swIAV vaccines. It is noteworthy, therefore, that group 3xBIV had an HI titer score of 2.5 against a recent H3N2 swIAV G19 (3.1970.1), which was isolated more than 40 years later than the vaccine strain and genetically distant, as shown by a P sequence value of 0.152. Three BIV administrations also induced an HI titer score ≥ 2 against pH1N1 (1A.3.3.2) IAVs (P sequence values 0.232–0.239), although no virus from the classical swine lineage (1A) was included in BIV. Another remarkable finding is the superior protection against the H1N1 challenge virus G18 (1C.2.1) in the 3xBIV group compared to the 3xTIV group. Still, the TIV H1N1 vaccine strain is genetically more closely related to the contemporary swIAVs including G18, than the BIV H1N1 vaccine strain (Figure 1; Additional files 1, 2, 3, 4). However, the vaccines did not only contain different virus strains, but also different adjuvants and antigen doses, although the antigenic mass cannot be directly compared since different units are used by manufacturers. All 3 factors have an impact on the vaccine immunogenicity [22, 63, 64] and in this study, the combination of these factors was probably more beneficial for group 3xBIV compared to group 3xTIV.

Besides anti-HA antibodies, the European commercial swIAV vaccines also induced an NI antibody response, which was more cross-reactive compared to the HI antibody response (Figures 4, 5). At the day of challenge, all vaccinated pigs had an NI titer score (≥ 1) against challenge virus G18. In group 3xMOV, only NI- but no HI antibodies were observed against G18. The high viral titers in the respiratory tract samples of this group suggest that the (limited) NI antibody response offered little or no protection, which is in accordance with previous pig vaccination studies [24, 65]. In the case of group 3xMOV, pigs were challenged with a heterologous swIAV of the same HA subtype as the vaccine strain, a situation that is believed to predispose for “vaccine-associated enhanced respiratory disease” (VAERD) [66]. However, in line with other European swine vaccination studies [18, 21–24, 52, 53], there were no significant differences in macroscopic lesion scores and histopathological findings among groups. As to group 3xMOV, the VAERD phenomenon might have been abrogated by the NI antibodies against G18 [67].

Unlike for human influenza viruses, it is impossible to select swIAV vaccine strains that would cover all of the circulating variants. This is due to the enormous genetic diversity of swIAVs, and one of the reasons why there is no formal system to recommend or update swIAV vaccine strains. In the field, swIAV vaccines are mainly used in sows rather than in fattening pigs. A preferred method is to vaccinate the gilts twice 2–4 weeks apart followed by a booster vaccination before each litter, to ensure higher and longer-lasting maternal antibody levels in the newborn piglet. While the booster vaccinations are traditionally performed with the same vaccine [18], our study shows that alternating doses of different commercial swIAV vaccines may help to broaden the antibody response. A number of questions need further investigation, such as the effect of longer time intervals between booster vaccinations, the duration of the induced immunity and whether there are more performant vaccine combinations. As an example, our data support the use of a multivalent vaccine (TIV or BIV), instead of the monovalent MOV vaccine, for the third vaccination, as this may boost antibody titers against H3 and human-like H1 swIAVs. Combinations of European and North American swIAV vaccines would results in an increased genetic and antigenic distance between vaccine strains and might also broaden the antibody responses [52, 53] (Chepkwony et al., unpublished observations). Finally, prime-boost regimens with a LAIV followed by a WIV booster were shown to result in higher T cell responses than 2 WIV administrations [31]. Thus, including novel generation vaccines, such as LAIV or vector vaccines, may further improve heterologous prime-boost vaccination strategies against swIAVs.

## Supplementary Information

**Additional file 1. P sequence values (upper right triangle) and P all antigenic site values (lower left triangle) for H1 of IAVs used in the study** [38, 39].

**Additional file 2. P sequence values (upper right triangle) and P all antigenic site values (lower left triangle) for H3 of IAVs used in the study **[40, 42].

**Additional file 3. P sequence values (upper right triangle) and P all antigenic site values (lower left triangle) for N1 of IAVs used in the study **[41].

**Additional file 4. P sequence values (upper right triangle) and P all antigenic site values (lower left triangle) for N2 of IAVs used in the study **[43].

## Data Availability

The data supporting the conclusions of this article are attached within the article and its additional files.
