# Supplementary material for: Alternating 3 different influenza vaccines for swine in Europe for a broader antibody response and protection
Source: Vet Res. 2022 Jun 15;53:44. doi: 10.1186/s13567-022-01060-x (PMC9202218; doi:10.1186/s13567-022-01060-x)
Supplement: Supplementary file 1 — Additional file 1. P sequence values (upper right triangle) and P all antigenic site values (lower left triangle) for H1 of IAVs used in the study [38, 39]. [file 13567_2022_1060_MOESM1_ESM.docx]

**Additional file 1. P sequence values (upper right triangle) and P all antigenic site values (lower left triangle) for H1 of IAVs used in the study [38, 39].**

|  |  |  | Vaccine strains | | | | | | | EU | | | | | | NA | | | Hu |
| --- | --- | --- | --- | --- | --- | --- | --- | --- | --- | --- | --- | --- | --- | --- | --- | --- | --- | --- | --- |
|  | Virus strains (HA clade) | GenBank accession number | HA03^TIV^  (1C.2.2) | *G05*  *(1C.2.1)* | BE83^BIV^*  (1C.1.2-like) | BA00^TIV^  (1B.1.2.1) | *G99*  *(1B.1.2.1)* | JE09^MOV^  (1A.3.3.2) | *CA09*  *(1A.3.3.2)* | **G18**  **(1C.2.1)** | NE19  (1C.2.2) | G19  (1B.1.2.1) | IT09  (1B.1.2.2) | ARM06  (1B.1.2.3) | G19  (1A.3.3.2) | IL10  (1B.2.2.2) | IL16  (1A.3.3.3) | SL15  (1A.3.3.2) | |
| Vax | HA03^TIV^ (1C.2.2) | GQ161119 |  | 0.034 | 0.141 | 0.291 | 0.296 | 0.288 | 0.272 | 0.083 | 0.098 | 0.309 | 0.296 | 0.317 | 0.281 | 0.276 | 0.260 | 0.288 | |
|  | *G05 (1C.2.1)* | CY116434 | 0.040 |  | 0.116 | 0.282 | 0.286 | 0.278 | 0.263 | 0.049 | 0.083 | 0.300 | 0.290 | 0.307 | 0.275 | 0.270 | 0.254 | 0.281 | |
|  | BE83^BIV^* (1C.1.2-like) | AF091316 | 0.240 | 0.200 |  | 0.249 | 0.261 | 0.239 | 0.232 | 0.122 | 0.162 | 0.281 | 0.265 | 0.282 | 0.232 | 0.255 | 0.211 | 0.239 | |
|  | BA00^TIV^ (1B.1.2.1) | GQ161104 | 0.580 | 0.580 | 0.600 |  | 0.016 | 0.291 | 0.279 | 0.288 | 0.304 | 0.077 | 0.093 | 0.085 | 0.298 | 0.148 | 0.267 | 0.298 | |
|  | *G99 (1B.1.2.1)* | AY590823 | 0.560 | 0.560 | 0.620 | 0.040 |  | 0.296 | 0.286 | 0.296 | 0.315 | 0.069 | 0.089 | 0.076 | 0.302 | 0.145 | 0.274 | 0.302 | |
|  | JE09^MOV^ (1A.3.3.2) | KC222636 | 0.380 | 0.360 | 0.420 | 0.660 | 0.640 |  | 0.028 | 0.294 | 0.288 | 0.303 | 0.287 | 0.295 | 0.052 | 0.295 | 0.119 | 0.034 | |
|  | *CA09 (1A.3.3.2)* | FJ966082 | 0.320 | 0.300 | 0.360 | 0.600 | 0.580 | 0.080 |  | 0.272 | 0.266 | 0.294 | 0.278 | 0.285 | 0.055 | 0.279 | 0.116 | 0.043 | |
| EU | **G18 (1C.2.1)** | EPI2026185 | 0.180 | 0.140 | 0.280 | 0.640 | 0.620 | 0.440 | 0.380 |  | 0.104 | 0.306 | 0.299 | 0.314 | 0.288 | 0.270 | 0.263 | 0.294 | |
|  | NE19 (1C.2.2) | MT395373 | 0.180 | 0.140 | 0.300 | 0.640 | 0.620 | 0.400 | 0.340 | 0.200 |  | 0.324 | 0.309 | 0.326 | 0.275 | 0.298 | 0.257 | 0.281 | |
|  | G19 (1B.1.2.1) | MW362630 | 0.600 | 0.600 | 0.660 | 0.180 | 0.140 | 0.660 | 0.600 | 0.640 | 0.660 |  | 0.120 | 0.107 | 0.312 | 0.160 | 0.291 | 0.309 | |
|  | IT09 (1B.1.2.2) | HM996939 | 0.640 | 0.640 | 0.680 | 0.220 | 0.200 | 0.660 | 0.620 | 0.680 | 0.660 | 0.280 |  | 0.114 | 0.287 | 0.164 | 0.278 | 0.290 | |
|  | ARM06 (1B.1.2.3) | AM503902 | 0.580 | 0.580 | 0.620 | 0.200 | 0.160 | 0.560 | 0.520 | 0.620 | 0.620 | 0.220 | 0.260 |  | 0.301 | 0.148 | 0.295 | 0.298 | |
|  | G19 (1A.3.3.2) | MW362718 | 0.400 | 0.380 | 0.440 | 0.660 | 0.640 | 0.120 | 0.120 | 0.440 | 0.400 | 0.680 | 0.640 | 0.580 |  | 0.282 | 0.135 | 0.028 | |
| NA | IL10 (1B.2.2.2) | JQ756323 | 0.540 | 0.540 | 0.600 | 0.360 | 0.340 | 0.540 | 0.500 | 0.560 | 0.600 | 0.380 | 0.360 | 0.320 | 0.560 |  | 0.291 | 0.295 | |
|  | IL16 (1A.3.3.3) | KU861208 | 0.380 | 0.360 | 0.340 | 0.620 | 0.600 | 0.220 | 0.180 | 0.380 | 0.340 | 0.620 | 0.660 | 0.620 | 0.260 | 0.560 |  | 0.119 | |
| Hu | SL15 (1A.3.3.2) | EPI223353 | 0.380 | 0.360 | 0.400 | 0.660 | 0.640 | 0.080 | 0.080 | 0.420 | 0.380 | 0.660 | 0.640 | 0.560 | 0.060 | 0.560 | 0.220 |  | |

The vaccine strains (TIV, Respiporc® FLU3; BIV, GRIPORK®; MOV, Respiporc® FLUpan H1N1) are abbreviated and the representative virus strain used for serology is shown in *italics* under each vaccine strain. The challenge virus is shown in **bold**. The HA virus clade is mentioned between brackets. The vaccine strains are shown first, followed by swine influenza A virus strains from Europe (EU), North America (NA) and human seasonal influenza A virus strains (Hu). See Figure 1 for full virus strain names.

P sequence is defined as: Number of amino acid substitutions in the HA1 domain of HA / Total number of amino acids in the HA1 domain of HA (327 amino acids).

P all antigenic site value is defined as: Number of amino acid substitutions in all 5 antigenic sites of the HA1 / Total number of amino acids in all 5 antigenic sites of the HA1 (50 amino acids) [38, 39]

*For A/swine/Olost/84, the avian-like H1N1 swIAV vaccine strain in BIV, no gene sequences are made publicly available. We therefore used an avian-like H1N1 swIAV (1C.2.1-like) from 1983 as a substitute for the genetic and serological investigations.
