# Supplementary material for: Alternating 3 different influenza vaccines for swine in Europe for a broader antibody response and protection
Source: Vet Res. 2022 Jun 15;53:44. doi: 10.1186/s13567-022-01060-x (PMC9202218; doi:10.1186/s13567-022-01060-x)
Supplement: Supplementary file 2 — Additional file 2. P sequence values (upper right triangle) and P all antigenic site values (lower left triangle) for H3 of IAVs used in the study [40, 42]. [file 13567_2022_1060_MOESM2_ESM.docx]

**Additional file 2. P sequence values (upper right triangle) and P all antigenic site values (lower left triangle) for H3 of IAVs used in the study [40, 42].**

|  |  |  | Vaccine strains | | | EU | NA | Hu |
| --- | --- | --- | --- | --- | --- | --- | --- | --- |
|  | Virus strains (HA clade) | GenBank accession number | BA03^TIV^  (3.1970.1) | *G00*  *(3.1970.1)* | PC73^BIV^  (3.1970.1) | G19  (3.1970.1) | MI15  (3.2010.1) | KA17  (huH3N2) |
| Vax | BA03^TIV^ (3.1970.1) | GQ161136 |  | 0.024 | 0.137 | 0.082 | 0.213 | 0.231 |
|  | *G00 (3.1970.1)* | KM822626 | 0.025 |  | 0.134 | 0.073 | 0.207 | 0.225 |
|  | PC73^BIV^ (3.1970.1) | CY113109 | 0.225 | 0.200 |  | 0.152 | 0.188 | 0.182 |
| EU | G19 (3.1970.1) | n.a. | 0.175 | 0.150 | 0.200 |  | 0.210 | 0.232 |
| NA | MI15 (3.2010.1) | KP901306 | 0.350 | 0.325 | 0.375 | 0.350 |  | 0.097 |
| Hu | KA17 (huH3N2) | MH080048 | 0.400 | 0.375 | 0.350 | 0.400 | 0.150 |  |

The vaccine strains (TIV, Respiporc® FLU3; BIV, GRIPORK®; MOV, Respiporc® FLUpan H1N1) are abbreviated and the representative virus strain used for serology is shown in *italics* under each vaccine strain. The challenge virus is shown in **bold**. The HA virus clade is mentioned between brackets. The vaccine strains are shown first, followed by swine influenza A virus strains from Europe (EU), North America (NA) and human seasonal influenza A virus strains (Hu). See Figure 1 for full virus strain names.

P sequence is defined as: Number of amino acid substitutions in the HA1 domain of HA / Total number of amino acids in the HA1 domain of HA (329 amino acids).

P all antigenic site is defined as: Number of amino acid substitutions in all 5 antigenic sites of the HA1 / Total number of amino acids in all 5 antigenic sites of the HA1 (40 amino acids) [40, 42].
