# Supplementary material for: Alternating 3 different influenza vaccines for swine in Europe for a broader antibody response and protection
Source: Vet Res. 2022 Jun 15;53:44. doi: 10.1186/s13567-022-01060-x (PMC9202218; doi:10.1186/s13567-022-01060-x)
Supplement: Supplementary file 3 — Additional file 3. P sequence values (upper right triangle) and P all antigenic site values (lower left triangle) for N1 of IAVs used in the study [41]. [file 13567_2022_1060_MOESM3_ESM.docx]

**Additional file 3. P sequence values (upper right triangle) and P all antigenic site values (lower left triangle) for N1 of IAVs used in the study [41].**

|  |  |  | Vaccine strains | | | | | EU | | | NA | Hu |
| --- | --- | --- | --- | --- | --- | --- | --- | --- | --- | --- | --- | --- |
|  | Virus strains (HA clade) | GenBank accession number | HA03^TIV^  (H1N1_av_) | *G05*  *(H1N1_av_)* | BE83^BIV*^  (H1N1_av_) | JE09^MOV^  (H1N1_pdm_) | *CA09*  *(H1N1_pdm_)* | **G18**  **(H1N1_av_)** | NE19  (H1N1_av_) | G19  (H1N1_pdm_) | IL16  (H1N1_classical_) | SL15  (H1N1_pdm_) |
| Vax | HA03^TIV^ (H1N1_av_) | GQ161121 |  | 0.036 | 0.060 | 0.081 | 0.077 | 0.060 | 0.060 | 0.098 | 0.181 | 0.094 |
|  | *G05 (H1N1_av_)* | CY116436 | 0.026 |  | 0.073 | 0.085 | 0.081 | 0.028 | 0.090 | 0.113 | 0.184 | 0.100 |
|  | BE83^BIV^* (H1N1_av_) | AJ412690 | 0.062 | 0.052 |  | 0.077 | 0.073 | 0.090 | 0.107 | 0.094 | 0.171 | 0.081 |
|  | JE09^MOV^ (H1N1_pdm_) | KX013007 | 0.073 | 0.062 | 0.073 |  | 0.004 | 0.100 | 0.120 | 0.051 | 0.169 | 0.032 |
|  | *CA09 (H1N1_pdm_)* | MK159404 | 0.067 | 0.057 | 0.067 | 0.005 |  | 0.096 | 0.115 | 0.051 | 0.169 | 0.032 |
| EU | **G18 (H1N1_av_)** | EPI2026186 | 0.052 | 0.031 | 0.062 | 0.067 | 0.062 |  | 0.103 | 0.122 | 0.186 | 0.107 |
|  | NE19 (H1N1_av_) | MT395375 | 0.041 | 0.057 | 0.083 | 0.093 | 0.088 | 0.062 |  | 0.126 | 0.190 | 0.124 |
|  | G19 (H1N1_pdm_) | MW362719 | 0.093 | 0.088 | 0.093 | 0.047 | 0.041 | 0.078 | 0.109 |  | 0.188 | 0.024 |
| NA | IL16 (H1N1_classical_) | KU861209 | 0.171 | 0.160 | 0.155 | 0.155 | 0.161 | 0.150 | 0.155 | 0.171 |  | 0.189 |
| Hu | SL15 (H1N1_pdm_) | EPI223353 | 0.093 | 0.083 | 0.083 | 0.031 | 0.026 | 0.067 | 0.109 | 0.026 | 0.166 |  |

The vaccine strains (TIV, Respiporc® FLU3; BIV, GRIPORK®; MOV, Respiporc® FLUpan H1N1) are abbreviated and the representative virus strain used for serology is shown in *italics* under each vaccine strain. The challenge virus is shown in **bold**. The NA virus lineage is mentioned between brackets. The vaccine strains are shown first, followed by swine influenza A virus strains from Europe (EU), North America (NA) and human seasonal influenza A virus strains (Hu). See Figure 1 for full virus strain names.

P sequence is defined as: Number of amino acid substitutions in the NA / Total number of amino acids in the NA (469 amino acids).

P all antigenic site is defined as: Number of amino acid substitutions in putative antigenic sites of the NA / Total number of amino acids in putative antigenic sites of the NA (193 amino acids) [41].

*For A/swine/Olost/84, the avian-like H1N1 swIAV vaccine strain in BIV, no gene sequences are made publicly available. We therefore used an avian-like H1N1 swIAV (1C.2.1-like) from 1983 as a substitute for the genetic and serological investigations.
