# Supplementary material for: Alternating 3 different influenza vaccines for swine in Europe for a broader antibody response and protection
Source: Vet Res. 2022 Jun 15;53:44. doi: 10.1186/s13567-022-01060-x (PMC9202218; doi:10.1186/s13567-022-01060-x)
Supplement: Supplementary file 4 — Additional file 4. P sequence values (upper right triangle) and P all antigenic site values (lower left triangle) for N2 of IAVs used in the study [43]. [file 13567_2022_1060_MOESM4_ESM.docx]

**Additional file 4. P sequence values (upper right triangle) and P all antigenic site values (lower left triangle) for N2 of IAVs used in the study [43].**

|  |  |  | Vaccine strains | | | | | EU | | | | NA | | Hu |
| --- | --- | --- | --- | --- | --- | --- | --- | --- | --- | --- | --- | --- | --- | --- |
|  | Virus strains (HA clade) | GenBank accession number | BA00^TIV^  (H1N2_swSC94_) | *G99*  *(H1N2_swSC94_)* | BA03^TIV^  (H3N2_swG84_) | *G00*  *(H3N2_swG84_)* | PC73^DIV^  (H3N2_swG84_) | G19  (H1N2_swSC94_) | IT09  (H1N2_it_) | ARM06  (H1N2_swSC94_) | G19  (H3N2_swSC94_) | IL10  (H1N2.2002) | MI15  (H1N2.2002) | KA17  (huH3N2) |
| Vax | BA00^TIV^ (H1N2_swSC94_) | JN088204 |  | 0.013 | 0.117 | 0.115 | 0.072 | 0.147 | 0.132 | 0.070 | 0.145 | 0.139 | 0.141 | 0.154 |
|  | *G99 (H1N2_swSC94_)* | AY590829 | 0.029 |  | 0.113 | 0.111 | 0.068 | 0.151 | 0.132 | 0.064 | 0.141 | 0.143 | 0.139 | 0.149 |
|  | BA03^TIV^ (H3N2_swG84_) | GQ161100 | 0.352 | 0.324 |  | 0.023 | 0.077 | 0.119 | 0.128 | 0.109 | 0.058 | 0.156 | 0.143 | 0.160 |
|  | *G00 (H3N2_swG84_)* | KR701539 | 0.294 | 0.265 | 0.059 |  | 0.068 | 0.102 | 0.126 | 0.104 | 0.051 | 0.154 | 0.139 | 0.156 |
|  | PC73^BIV^ (H3N2_swG84_) | CY009350 | 0.235 | 0.206 | 0.235 | 0.176 |  | 0.117 | 0.066 | 0.098 | 0.102 | 0.115 | 0.113 | 0.130 |
| EU | G19 (H1N2_swSC94_) | MW362632 | 0.412 | 0.441 | 0.382 | 0.324 | 0.382 |  | 0.143 | 0.147 | 0.134 | 0.151 | 0.143 | 0.192 |
|  | IT09 (H1N2_it_) | HM996952 | 0.324 | 0.353 | 0.412 | 0.382 | 0.412 | 0.353 |  | 0.119 | 0.151 | 0.090 | 0.091 | 0.102 |
|  | ARM06 (H1N2_swSC94_) | KR701505 | 0.147 | 0.118 | 0.294 | 0.235 | 0.176 | 0.471 | 0.412 |  | 0.119 | 0.124 | 0.124 | 0.139 |
|  | G19 (H3N2_swSC94_) | n.a. | 0.324 | 0.294 | 0.088 | 0.029 | 0.206 | 0.353 | 0.412 | 0.206 |  | 0.171 | 0.158 | 0.171 |
| NA | IL10 (H1N2.2002) | JQ756349 | 0.441 | 0.471 | 0.588 | 0.588 | 0.500 | 0.471 | 0.353 | 0.471 | 0.559 |  | 0.070 | 0.105 |
|  | MI15 (H3N2.2002) | KP901307 | 0.412 | 0.382 | 0.471 | 0.441 | 0.412 | 0.412 | 0.265 | 0.441 | 0.471 | 0.324 |  | 0.113 |
| Hu | KA17 (huH3N2) | MH600790 | 0.412 | 0.382 | 0.471 | 0.471 | 0.412 | 0.529 | 0.353 | 0.382 | 0.441 | 0.235 | 0.206 |  |

The vaccine strains (TIV, Respiporc® FLU3; BIV, GRIPORK®; MOV, Respiporc® FLUpan H1N1) are abbreviated and the representative virus strain used for serology is shown in *italics* under each vaccine strain. The challenge virus is shown in **bold**. The NA virus lineage is mentioned between brackets. The vaccine strains are shown first, followed by swine influenza A virus strains from Europe (EU), North America (NA) and human seasonal influenza A virus strains (Hu). See Figure 1 for full virus strain names.

P sequence is defined as: Number of amino acid substitutions in the NA / Total number of amino acids in the NA (469 amino acids).

P all antigenic site is defined as: Number of amino acid substitutions in putative antigenic sites of the NA / Total number of amino acids in putative antigenic sites of the NA (34 amino acids) [43]
